# Mast Cell Population and Histamine Content in Hypothyroid Rat Tissues

**DOI:** 10.3390/ani12141840

**Published:** 2022-07-20

**Authors:** Gabriella Chieffi Baccari, Sara Falvo, Antonia Lanni, Maria Maddalena Di Fiore, Federica Cioffi, Alessandra Santillo

**Affiliations:** 1 Department of Environmental, Biological and Pharmaceutical Sciences and Technologies, University of Campania “Luigi Vanvitelli”, 81100 Caserta, Italy; sara.falvo@unicampania.it (S.F.); antonia.lanni@unicampania.it (A.L.); mariamaddalena.difiore@unicampania.it (M.M.D.F.); alessandra.santillo@unicampania.it (A.S.); 2 Department of Science and Technology, University of Sannio, 82100 Benevento, Italy; fecioffi@unisannio.it

**Keywords:** mast cells, histamine, thyroid hormones, hypothyroidism, skin, exorbital lacrimal gland

## Abstract

**Simple Summary:**

In this study, we investigated the putative regulatory role of the pituitary–thyroid axis on mast cells (MCs). We found that hypothyroidism resulted in a significant increase in number of MCs and the histamine content in the skin and exorbital lacrimal gland of Wistar rats, *Rattus norvegicus*. Furthermore, an increase in the percentage of degranulating MCs suggests that the thyroid status also influences the activation state of these cells.

**Abstract:**

The morphological features and relative number of mast cells (MCs) were studied in the skin and exorbital lacrimal glands of hypothyroid Wistar rats, *Rattus norvegicus*. Hypothyroidism significantly increased the number of MCs (up to 4.5-fold) and histamine content (up to 50%) in the examined tissues. The magnitude of the increase in the number of MCs was greater in the cheek skin and exorbital lacrimal glands than in the back skin. In the skin, the MCs were mainly located within the hypodermis and closely associated with the blood vessels, nerve fascicles, and adipocytes. In the exorbital lacrimal gland, which is a seromucous gland located lateral to the cheek below the ear, the MCs were distributed in the connective tissue surrounding the acini. The secretory granules of MCs showed histochemical characteristics of connective tissue MCs. They were metachromatic with Toluidine blue and safranin positive with the Alcian blue/safranin reactions. Finally, a significant increase in degranulating MCs was observed in hypothyroid tissues, relative to euthyroid tissues. At the ultrastructural level, the MCs of euthyroid rats were predominantly non-degranulating (Stage I). In hypothyroid animals, numerous MCs showed partial degranulation (Stage II–III) or were in a stage of complete degranulation. Our results concerning the skin and exorbital lacrimal gland suggested that the thyroid status might be involved in regulating the frequency and activation state of MCs.

## 1. Introduction

Mast cells (MCs) are the only tissue-resident cells that possess high affinity receptors for IgE and synthesize histamine [1]. Although they are now considered to be multifunctional immune cells that are implicated in various health and disease states, the wide “popularity” of MCs in mammalian tissues is attributable to their main role in immune response [2,3]. 

MCs originate from bone marrow and differentiate after invading tissues, as they are influenced by the local environment [4]. MCs are widely distributed in the tissues that are the potential sites of pathogen entry, such as the respiratory mucosa, gastrointestinal tract, and skin [5,6,7]. Skin MCs populate the dermis and hypodermis, where they are located predominantly near blood vessels and nerve fascicles, as well as in the connective tissue surrounding glandular acini. 

The cytoplasm of MCs is filled with secretory granules containing histamine, heparin, other vasoactive amines, and proteases [8]. Proinflammatory lipid mediators, such as prostaglandins, leukotrienes, and hydroxyeicosatetraenoic acids, are produced de novo and released upon stimulation [9]. However, the composition of the granules of MCs shows some heterogeneity, depending on their tissue localization [7]. Two major subtypes of MCs, including connective tissue and mucosal, have been identified in rodents [10]. These subtypes can be differentiated by their histological, biochemical, functional, and pharmacological properties [11]. Recently, anatomically distinct subtypes of mouse MCs were identified through transcriptomic analysis [12].

Studies regarding the regulation of MCs via thyroid hormones are scarce, and data are based primarily on observations in a few organs under specific endocrine conditions. Administration of an anti-thyroid agent, 6-n-propyl-2-thiouracil (PTU), increases the number of MCs in the neonatal rat brain, whereas treatment with thyroid (3,5,3-triiodothyronine or thyroxine) or thyrotropic (TSH) hormones at up to five days of age induces a reduction of MC population in the brain [13]. A higher number of MCs was also observed in the bone marrow of hypothyroid rats than in euthyroid, thyrotoxic, or hypothyroxine-substituted animals [14]; this occurred due to the expression of T3 receptors α1 (TRα1), TRα2, and TRβ1 in the MCs of rat bone tissue. Although clinical evidence has, for decades, indicated that cutaneous MCs increase in humans under hypothyroidism (for review, see [15,16]), few experimental studies have been performed to elucidate the role of thyroid hormones as modulators of MC population and activators of histamine release. Here, we showed the effects of the hypothyroid status on MC population of the skin and, for the first time, in the exorbital lacrimal glands of rats. This short communication highlights a useful experimental model, in which hypothyroidism was induced by a chemical treatment that produces severe hypothyroidism and inhibition of the three known types of deiodinase enzymes. Using this approach, it is possible to exclude that the observed effect is due to a deiodination products. 

## 2. Materials and Methods

### 2.1. Animals and Experiments

Male Wistar rats, *Rattus norvegicus albinus* (body weight: 300–350 g), were fed commercial pellets (Mil-Rat, Morini, Italy) and water ad libitum; they were kept under regulated temperature and light conditions. Rats (N = 8) received daily intraperitoneal injections (i.p.) of 6-n-propylthiouracil (PTU) (1 mg/100 g body mass) for 4 consecutive weeks; once a week, they received an i.p. injection of iopanoic acid (IOPA) (6 mg/100 g body mass) [17]. PTU blocks thyroid hormone synthesis via inhibition of thyroid peroxidase activity, and it is also a strong inhibitor of type I 5′-D-deiodinase activity. IOPA inhibits all three types of deiodinase enzymes (I, II, and III). Control rats (N = 8) received saline injections. At the end of the treatment, the rats were sacrificed, and the trunk blood was collected to determine T3 and TSH levels. Samples of shaved skin from both cheek and back, as well as the exorbital lacrimal glands, were dissected out, weighed, and either used for histamine determination or rapidly immersed in fixative for microscopy analysis [17]. 

Animal care and experimental procedures were conducted in accordance with the guidelines of the Ethics Committee of the University of Campania “Luigi Vanvitelli” and Italian Minister of Health. Every effort was made to minimize animal pain and suffering. 

### 2.2. T3 and TSH Serum Determinations

Serum total 3,5,3-triiodothyronine (T3) levels were quantified according to protocols provided by BD Biosciences (Franklin Lakes, NJ, USA). Serum TSH levels were determined by radioimmunoassay (RIA), according to protocols provided by Amersham (Milan, Italy) [17]. 

### 2.3. Histochemistry and Ultrastructure

For histochemical analysis pieces (5–6 mm^3^) of skin and exorbital lacrimal gland from each animal (*n* = 8 for each experimental group) were embedded in paraffin. Serial paraffin sections (5 µm-thick) were stained with (1) 0.2% Toluidine blue in Walpole buffer at pH 4.2; (2) Alcian blue/safranin (AB/safranin) [18].

For electron microscopy, pieces of skin and exorbital lacrimal glands (3 mm^3^) were immersed in Karnovsky’s fixative in cacodylate buffer (pH 7.4) and then postfixed for 2 h in cacodylate buffer containing 1% osmium tetroxide [19]. Sections were examined using a Zeiss LEO-912 transmission electron microscope.

### 2.4. Morphometric Analysis

The number of MC/mm^2^ and percentage of degranulating MCs were determined by digitization of Toluidine blue-stained sections (twenty randomly chosen from each experimental sample) viewed under a Nikon Eclipse E600 light microscope (Melville, NY, USA) with an attached JVC TK-C1381 photo camera (Tokyo, Japan) connected to a Pentium II computer (Intel Corp., Assago, Italy) running Lucia ScMeas on Mutech software (Fairfield, NJ, USA). The data were checked for normal distribution by using a histogram test. The data, expressed as means ± standard deviation (SD), were compared by Student’s *t*-test for between-group comparisons. The level of significance was taken at *p* < 0.05.

### 2.5. Histamine Determination

For the determination of histamine content, pieces of skin (from the cheek and back) and exorbital lacrimal glands were dehydrated on filter paper, weighed, and boiled in 8% HClO_4_ for 30 min. Fluorimetric method was used for determination of histamine levels. All values are based on the means of duplicate determinations. The data were checked for normal distribution by using a histogram test. Student’s t test for between-group comparisons was used to compare the values, which were expressed as mean ± S.D. The significance level was assumed to be *p* < 0.05.

## 3. Results

PTU and IOPA-treated rats showed T3 levels significantly lower (0.13 ± 0.02 nmol/L) than those observed in euthyroid rats (0.98 ± 0.05 nmol/L) [17]. The serum TSH level was considerably higher in hypothyroid rats (20.1 ± 1.2 mIU/L), compared to that in euthyroid rats (4.3 ± 0.4 mIU/L) [17].

**Figure 1:**
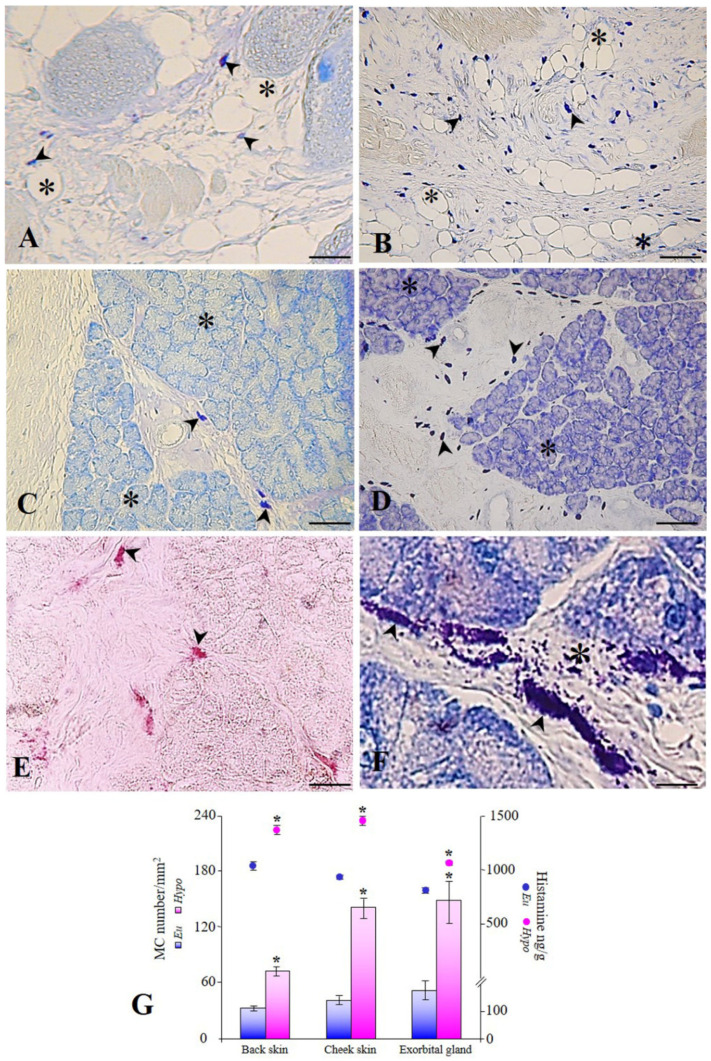
(**A**) Metachromatic MCs (arrowheads) among adipocytes (asterisks) in the hypodermis of euthyroid rats. Scale bar, 150 µm. (**B**) Numerous metachromatic MCs (arrowheads) among adipocytes (asterisks) in the hypodermis of hypothyroid rats. Scale bar, 300 µm. (**C**) Metachromatic MCs (arrowheads) among acini (asterisks) in the exorbital glands of euthyroid rats. Scale bar, 250 µm. (**D**) Numerous MCs (arrowheads) in the connective tissue among acini (asterisks) in the exorbital glands of hypothyroid rats. Scale bar, 300 µm. (**E**) Safranin positive MCs (arrowheads) in the exorbital glands of hypothyroid rats. Scale bar, 75 µm. (**F**) Degranulating MCs (arrowheads) in the exorbital glands of hypothyroid rats. Note the numerous granules released into the tissue microenvironment (asterisk). Scale bar, 20 µm. (**G**) MCs number/mm^2^ and histamine ng/g tissue in euthyroid and hypothyroid rat tissues. * *p* < 0.05. (**A**–**D**,**F**) Toluidine blue at pH 4.2; (**E**) AB/safranin staining.

In hypothyroid animals, the number of MCs/mm^2^ was significantly higher in the back skin (73 ± 13/mm^2^), cheek skin (141 ± 26/mm^2^), and exorbital lacrimal glands (149 ± 41/mm^2^), compared to that in euthyroid tissues (back skin: 33 ± 11/mm^2^; cheek skin: 41 ± 14/mm^2^; exorbital lacrimal gland: 52 ± 18/mm^2^) (Figure 1G). In the back and cheek skin of euthyroid (Figure 1A) and hypothyroid rats (Figure 1B), the MCs were observed mainly in the hypodermis, which contains more numerous adipocytes, larger blood vessels, and nerves than those found in the dermis. MCs were closely associated with adipocytes, blood vessels, and nerve fascicles. In the exorbital lacrimal glands, an exocrine seromucous glands located laterally to the cheek below the ear [20], the MCs were present in the connective tissue surrounding the acini (Figure 1C,D). In the examined tissues from experimental groups, the MCs contain granules that stained metachromatically with Toluidine blue (Figure 1A–D,F) and red with the sequential AB/safranin reaction (Figure 1E). AB/safranin reaction distinguishes the granules containing low-sulphated glycosaminoglycans (AB-positive) from the granules containing high-sulphated glycosaminoglycans (safranin-positive). The metachromasia and the safranin positivity indicated the presence of highly sulphated glycosaminoglycans, such as heparin. 

A fluorimetric method was used to determine the tissue histamine content. The results showed that the histamine content in the hypothyroid tissues (back skin: 1358 ng/g; cheek skin: 1436 ng/g; exorbital lacrimal gland: 1069 ng/g) was higher than that in the euthyroid tissues (back skin: 1037 ng/g; cheek skin: 937 ng/g; exorbital lacrimal gland: 818 ng/g) (Figure 1G). 

**Figure 2:**
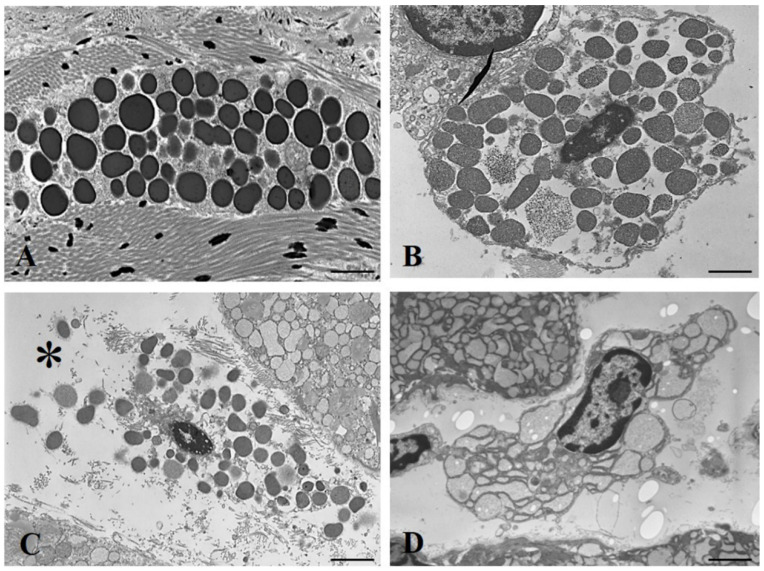
(**A**) Electron micrograph of a cutaneous mast cell surrounded by collagen fibers from euthyroid rat. The cytoplasm contains numerous electron-dense granules. (**B**) A mast cell (Stage II) in the exorbital gland from hypothyroid rat; (**C**) a mast cell (Stage III) in the exorbital gland from hypothyroid rat. Note the numerous granules released into the tissue microenvironment (*). (**D**) A fully degranulating mast cell in the exorbital gland from hypothyroid rat. (**A**–**D**) Scale bars, 5 µm (original magnification).

The secretory cycle of MCs includes a resting state and state of secretory activity, in which chemical mediators are released in the surrounding microenvironment [21]. We observed a higher percentage of degranulating MCs in the back skin (28%), cheek skin (32%), and exorbital lacrimal glands (39%) of hypothyroid rats than that in euthyroid rats (10%, 18%, and 19%, respectively). The degranulating MCs showed numerous extracellular metachromatic granules and/or a poor content of intracytoplasmic granules (Figure 1F). At the ultrastructural level, the secretory cycle of MCs can be divided into the following stages: Stage I: resting MCs showing a cytoplasm filled with electron-dense granules; Stage II: the initiation of degranulation, in which large granules with heterogeneous electron density are observed; Stage III: the appearance of fine particulate material and release of granules in the tissue microenvironment; finally, a stage of complete degranulation, followed by a stage of regeneration [19]. We found that the MCs from euthyroid rats were predominantly at Stage I (Figure 2A). They were elongated in shape, with a cytoplasm filled with homogeneously osmiophilic secretory granules. In hypothyroid animals, numerous MCs in Stages II (Figure 2B) and III (Figure 2C), or fully degranulated (Figure 2D), were observed; MCs were rarely detected in the regenerative stage.

## 4. Discussion

We found a significant increase in the number of MCs and histamine content in the hypothyroid tissues, compared to that in the euthyroid tissues. The magnitude of increase in the number of MCs was greater in the cheek skin and exorbital lacrimal glands than in the back skin. Particularly, in hypothyroid rats, the number of MCs was higher by about 4.5-fold in the cheek skin, 3.5-fold in the exorbital gland, and 1.5-fold in the back skin, relative to that in the euthyroid rats. 

The histamine content in hypothyroid animals was higher by about 50% in the cheek skin, 25% in the exorbital lacrimal gland, and 32% in the back skin. Therefore, although a drastic increase in MC number occurred in hypothyroid rats, the increase in histamine content was moderate. This inconsistency could indicate that a majority of tissue MCs are immature cells containing low histamine levels. Histamine is mainly a pro-inflammatory mediator involved in local inflammation, itch, and vasodilation. Additionally, MCs have histaminergic receptors that enable histamine to exert paracrine control over MC degranulation [22]. The morphological study showed that the MCs in hypothyroid rats mostly infiltrated the hypodermis, where they were closely associated with adipocytes, blood vessels, and nerve fascicles. In the exorbital lacrimal glands, numerous MCs were observed in the connective tissue surrounding the acini. The histochemical analysis indicated that the infiltrating MCs were connective tissue-type MCs. The ultrastuctural analysis showed a higher percentage of partially and/or fully degranulating MCs (Stages II and III) in the hypothyroid tissues. The release of secretory granules from MCs in the tissue microenvironment could likely be induced by the paracrine action of histamine [22].

In line with these results, the infiltration of MCs has been described in the liver of hypothyroid rabbits [23]. Additionally, a large numbers of MCs were observed in the bone marrow of hypothyroid rats, compared to that of euthyroid and hypothyroid-thyroxine replaced animals [14]. MCs in rat bone tissue express T3 receptors (TRα1, TRα2, and TRβ1) [14], and peritoneal MCs contain T3, whose concentration is regulated by TSH [24]. These facts indicate that thyroid hormones might play a regulatory role in the dynamics of MCs, and MC degranulation might affect thyroid function; the latter aspect needs to be validated [16]. 

Some authors [25,26,27,28] reported an increase in the number of MCs in the thyroid gland, following the administration of anti-thyroid drugs, and speculated that these effects might be due to an increase in the circulating TSH levels elicited by anti-thyroid agents. Therefore, it could be hypothesized that the increase in the number of MCs detected in the skin and exorbital glands of PTU/IOPA-treated rats could be due to high levels of TSH. In another study, we showed that PTU treatment inflicts an increase of MC number in the brain, while hipophysectomy induces a drastic reduction in the population of MCs [29]. The negative effect of hipophysectomy is effectively counteracted by replacement therapy with homologous pars distalis homogenate [29]. In the harderian gland, the pituitary gland influences the population of MCs; among pituitary hormones, TSH and adrenocorticotropic hormone (ACTH) are the most influential [30].

## 5. Conclusions

To summarize, we provided experimental evidence regarding the infiltration of MCs in the skin and exorbital gland connective tissues with the consequent increase in tissue histamine levels in hypothyroid rats. The number of MCs and histamine content increased by up to 4.5-fold and 50%, respectively. Finally, an increase in the percentage of degranulating MCs suggests that the thyroid status influences the activation of these cells. Further investigations are needed, in order to clarify the action mechanism of pituitary–thyroid axis in the modulation of the MC population and activation of histamine release.

## Data Availability

The data presented in this study are available on request from the corresponding author.

## References

[1] Galli S.J., Tsai M. (2012). IgE and mast cells in allergic disease. Nat. Med..

[2] da Silva E.Z., Jamur M.C., Oliver C. (2014). Mast cell function: A new vision of an old cell. J. Histochem. Cytochem..

[3] Varricchi G., de Paulis A., Marone G., Galli S.J. (2019). Future Needs in Mast Cell Biology. Int. J. Mol. Sci..

[4] Kitamura Y. (1989). Heterogeneity of mast cells and phenotypic change between subpopulations. Ann. Rev. Immunol..

[5] Galli S.J., Nakae S., Tsai M. (2005). Mast cells in the development of adaptive immune responses. Nat. Immunol..

[6] Metcalfe D.D., Boyce J.A. (2006). Mast cell biology in evolution. J. Allergy Clin. Immunol..

[7] Baccari G.C., Pinelli C., Santillo A., Minucci S., Rastogi R.K. (2011). Mast cells in nonmammalian vertebrates: An overview. Int. Rev. Cell. Mol. Biol..

[8] Beil W.J., Schulz M., Wefelmeyer U. (2000). Mast cell granule composition and tissue location—A close correlation. Histol. Histopathol..

[9] Nakamura T. (2021). The roles of lipid mediators in type I hypersensitivity. J. Pharmacol. Sci..

[10] Tainsh K.R., Pearce F.L. (1992). Mast Cell Heterogeneity: Evidence that Mast Cells Isolated from Various Connective Tissue Locations in the Rat Display Markedly Graded Phenotypes. Int. Arch. Allergy Immunol..

[11] Bienenstock J. (1988). An update on mast cell heterogeneity. J. Allergy Clin. Immunol..

[12] Akula S., Paivandy A., Fu Z., Thorpe M., Pejler G., Hellman L. (2020). Quantitative In-Depth Analysis of the Mouse Mast Cell Transcriptome Reveals Organ-Specific Mast Cell Heterogeneity. Cells.

[13] Sabria J., Ferrer I., Toledo A., Sentis M., Blanco I. (1987). Effects of altered thyroid function on histamine levels and mast cell number in neonatal rat brain. J. Pharmacol. Exp. Ther..

[14] Siebler T., Robson H., Bromley M., Stevens D.A., Shalet S.M., Williams G.R. (2002). Thyroid status affects number and localization of thyroid hormone receptor expressing mast cells in bone marrow. Bone.

[15] Artantaş Ş., Gül Ü., Kılıç A., Güler S. (2009). Skin findings in thyroid diseases. Eur. J. Intern. Med..

[16] Landucci E., Laurino A., Cinci L., Gencarelli M., Raimondi L. (2019). Thyroid Hormone, Thyroid Hormone Metabolites and Mast Cells: A Less Explored Issue. Front. Cell. Neurosci..

[17] Monteforte R., Santillo A., Lanni A., D’Aniello S., Baccari G.C. (2008). Morphological and biochemical changes in the Harderian gland of hypothyroid rats. J. Exp. Biol..

[18] Ribatti D. (2018). The Staining of Mast Cells: A Historical Overview. Int. Arch. Allergy Immunol..

[19] Baccari G.C., De Paulis A., Di Matteo L., Gentile M., Marone G., Minucci S. (1998). In situ characterization of mast cells in the frog Rana esculenta. Cell Tissue Res..

[20] Ferrara D., Monteforte R., Baccari G.C., Minucci S., Chieffi G. (2004). Androgen and estrogen receptors expression in the rat exorbital lacrimal gland in relation to “harderianization”. J. Exp. Zool. Part A Comp. Exp. Biol..

[21] Wilhelm M., King B., Silverman A.J., Silver R. (2000). Gonadal steroids regulate the number and activational state of mast cells in the medial habenula. Endocrinology.

[22] Thangam E.B., Jemima E.A., Singh H., Baig M.S., Khan M., Mathias C.B., Church M.K., Saluja R. (2018). The Role of Histamine and Histamine Receptors in Mast Cell-Mediated Allergy and Inflammation: The Hunt for New Therapeutic Targets. Front. Immunol..

[23] Rodríguez-Castelán J., Corona-Pérez A., Nicolás-Toledo L., Martínez-Gómez M., Castelán F., Cuevas-Romero E. (2017). Hypothyroidism Induces a Moderate Steatohepatitis Accompanied by Liver Regeneration, Mast Cells Infiltration, and Changes in the Expression of the Farnesoid X Receptor. Exp. Clin. Endocrinol. Diabetes.

[24] Csaba G., Pállinger É. (2009). Thyrotropic hormone (TSH) regulation of triiodothyronine (T3) concentration in immune cells. Inflamm. Res..

[25] Clayton J.A., Masuoka D.T. (1968). TSH-Induced Mobilization of Serotonin from Perivascular Mast Cells in the Rat Thyroid. Endocrinology.

[26] Ericson L.E., Håkanson R., Melander A., Owman C., Sundler F. (1972). TSH-Induced Release of 5-Hydroxytryptamine and Histamine from Rat Thyroid Mast Cells. Endocrinology.

[27] Melander A., Sundler F. (1972). Significance of Thyroid Mast Cells in Thyroid Hormone Secretion. Endocrinology.

[28] Wynford-Thomas D., Stringer B.M.J. (1982). Mast cell hyperplasia during goitrogen—Induced thyroid growth—A quantitative study. Eur. J. Endocrinol..

[29] Monteforte R., Pinelli C., Santillo A., Rastogi R.K., Polese G., Baccari G.C. (2010). Mast cell population in the frog brain: Distribution and influence of thyroid status. J. Exp. Biol..

[30] Baccari G.C., Minucci S., Marmorino C., Izzo I.V. (1991). Number of mast cells in the harderian gland of the green frog, Rana esculenta: The annual cycle and its relation to environmental and hormonal factors. J. Anat..

